# Supplementary material for: Non-target impacts of fungicide disturbance on phyllosphere yeasts in conventional and no-till management
Source: ISME Commun. 2022 Feb 23;2:19. doi: 10.1038/s43705-022-00103-w (PMC9674006; doi:10.1038/s43705-022-00103-w)
Supplement: Supplementary file 1 — Supplementary information [file 43705_2022_103_MOESM1_ESM.docx]

**Supplementary Methods**

**Fungicide Applications**

In 2017, the fungicide Headline^®^ was applied to maize foliage at a recommended rate of 877 ml ha^-1^ (12 fl oz acre^-1^). Headline^®^ contains the QoI active ingredient pyraclostrobin, which acts as a mitochondrial respiration inhibitor. Pyraclostrobin is a local penetrant fungicide with translaminar movement and is not translocated in the xylem [[1]](https://paperpile.com/c/QdsGZD/oZ0M). In 2018, soybean foliage was sprayed with Delaro^®^ fungicide on 3 August 2018 (Bayer, Raleigh, NC, USA) at a recommended rate of 731 ml ha^-1^ (11 fl oz acre^-1^). The active ingredients in Delaro^®^ are a combination of the QoI trifloxystrobin which inhibits mitochondrial respiration, and DMI prothioconazole, which inhibits ergosterol synthesis. Trifloxystrobin is a local penetrant fungicide with translaminar movement and is not translocated in the xylem [[1]](https://paperpile.com/c/QdsGZD/oZ0M). Prothioconazole has acropetal penetrant activity and has weak basipetal movement [[2]](https://paperpile.com/c/QdsGZD/y8hp).

**Sample Collection and DNA Extraction**

Maize leaves were sampled by removing two whole leaves from each plant and placing them into a sterile Whirl Pak (Nasco, Madison, WI, USA) for transport back to the lab where they were stored at -80ºC until they were lyophilized. At the V6 and V8 growth stage, the sixth and seventh leaf was sampled. However, at the V15 growth stage, three leaves above the ear leaf were sampled. Roots were sampled by removing whole plants from the soil and the entire root system to the soil line. Then roots were washed in the field before being transported back to the lab, where roots were washed again with 0.1% tween 20 (ThermoFisher Scientific, USA) and deionized water. Samples were stored at -80ºC before being lyophilized for DNA extraction. Following lyophilization, the fine roots were removed from the root system and used for DNA extraction.

Soybean leaves were sampled with a flamed metal hole punch, washed in 80% ethanol, and flame sterilized between samples. Three 6-mm leaf discs from three separate leaves were punched directly into an Eppendorf tube (Eppendorf, Germany) containing 500 μl of CSPL buffer (Omega Bio-Tek, Norcross, GA, USA). As with the maize roots, whole soybean plants were removed from soil and soybean roots were removed at the soil line and placed into a new Whirl-Pak (Nasco, Madison, WI, USA) bag containing approximately 50 ml of 0.1% tween 20 to remove the remaining soil. Root samples were transported back to the lab, where roots were washed again with DI water, and samples were stored at -80ºC until processing. Maize and soybean leaf and root tissue were pulverized for 2-min at a speed of 30 Hz with two 4-mm stainless balls in a TissueLyser II (Qiagen, Venlo, Netherlands). Total DNA was extracted from plant tissues with the OMEGA Mag-Bind Plant DNA Plus kit (Omega Bio-Tek, Norcross, GA, USA) following the manufacturer’s instructions with the aid of a KingFisher Flex^TM^ liquid handling machine (ThermoFisher Scientific, USA). Five or six internal negative extraction controls were included per 96-well plate in each DNA extraction.

**Amplicon library preparation for ITS and 16s community profiling.** Amplicon libraries were prepared from a modified three-step PCR protocol as described previously [[3, 4]](https://paperpile.com/c/QdsGZD/M2TK+Ozm7). In brief, fungal libraries were constructed around the ITS and were amplified using the primers ITS1F and ITS4 [[5]](https://paperpile.com/c/QdsGZD/48PI). Prokaryote libraries targeted the V4 region of 16S rRNA with the primers 515F and 806R [[6]](https://paperpile.com/c/QdsGZD/CNhY). Supporting information Tables S1, S2 and S3 describe PCR protocols, primers, and cycling conditions in detail. Amplicons were purified with the SequalPrepTM Normalization Plate Kit (ThermoFisher Scientific, USA) and then pooled and concentrated with Amicon® Ultra 0.5 mL filters (EMDmillipore, Germany). Subsequently, the library was purified, and size selected with Agencourt AMPure XP magnetic beads (Beckman Coulter, USA). Amplicon libraries were quantified and checked on the Agilent 4200 TapeStation DNA10000 and Kapa Illumina Library Quantification qPCR assays. All amplicon libraries were then paired-end sequenced (300 bp reads) on an Illumina MiSeq with a v3 600 cycles kit (Illumina, USA).

**Bioinformatic sequence processing.** Fungal ITS1 or prokaryotic 16S V4 reads were demultiplexed in QIIME 1.9.1 [[7]](https://paperpile.com/c/QdsGZD/ZviC). Forward and reverse prokaryote reads were merged using QIIME 1.9.1. Only forward fungal ITS1 reads were used since reverse reads did not overlap. After removing primers with Cutadapt 1.8.1 [[8]](https://paperpile.com/c/QdsGZD/4GSD), fungal reads were trimmed to remove the conserved SSU and 28S regions. Reads were then quality filtered at an expected error threshold of 0.1 and truncated to equal length (fungi 200 bp; prokaryote 300 bp) in USEARCH 11.0.667 [[9]](https://paperpile.com/c/QdsGZD/x7HG). Singletons and chimeras were removed, and *de novo* OTU clustering was performed at a 97% similarity using the UPARSE algorithm [[10]](https://paperpile.com/c/QdsGZD/E01p). Using CONSTAX2 [[11, 12]](https://paperpile.com/c/QdsGZD/6D96+ufZK), the taxonomic classification of fungal and prokaryotic OTU's representative sequences was performed against the UNITE eukaryote database, ver. 8.2 of 04.02.2020 [[13]](https://paperpile.com/c/QdsGZD/0nFE) and SILVA, version 138 [[14]](https://paperpile.com/c/QdsGZD/Bhhe), respectively. To filter out non-target taxa and OTUs unidentified at the Kingdom level, CONSTAX was run twice under different cutoff levels, as suggested by Bowsher *et al.* (2020) [[15]](https://paperpile.com/c/QdsGZD/gKuO). Non-target taxa, OTUs not assigned to a Kingdom, and OTUs identified as either chloroplast or mitochondria in either database were removed from further analysis [[16]](https://paperpile.com/c/QdsGZD/xDpe).

**Import and Preprocessing in R.** Data were imported into R 4.0.3 [[17]](https://paperpile.com/c/QdsGZD/LUeU), and the R packages *phyloseq* 1.24.2 [[18]](https://paperpile.com/c/QdsGZD/WA6X) and *vegan* 2.5.3 [[19]](https://paperpile.com/c/QdsGZD/kSOr) were used for most analyses. Samples with low sequencing coverage (less than 1000 reads) were removed from the analysis. Contaminant OTUs (i.e., those prevalent in negative extraction controls) were removed with the R package *decontam* [[20]](https://paperpile.com/c/QdsGZD/udS7). Before normalization, richness was assessed for Prokaryotes and Fungi in the leaves and roots of each crop using the ‘estimate_richness’ function in the *phyloseq* package. Results of alpha diversity analyses were plotted using the ‘ggplot2’ package [[21]](https://paperpile.com/c/QdsGZD/04mL). Then, sample read counts were normalized using the cumulative sum scaling technique within the *metagenomeSeq* R package [[22]](https://paperpile.com/c/QdsGZD/xmNQ).

**References**

1. [Latin R. A Practical Guide to Turfgrass Fungicides. (APS Publications, 2017).](http://paperpile.com/b/QdsGZD/oZ0M)

2. [Augusto J, Brenneman TB. Assessing Systemicity of Peanut Fungicides Through Bioassay of Plant Tissues with Sclerotium rolfsii. *Plant Dis*](http://paperpile.com/b/QdsGZD/y8hp). [2012; **96**: 330–337.](http://paperpile.com/b/QdsGZD/y8hp)

3. [Longley R, Noel ZA, Benucci GMN, Chilvers MI, Trail F, Bonito G. Crop Management Impacts the Soybean (Glycine max) Microbiome. *Frontiers in Microbiology* . 2020; **11**](http://paperpile.com/b/QdsGZD/M2TK)**.**

4. [Fehr WR, Caviness CE, Burmood DT, Pennington JS. Stage of development descriptions for soybeans, Glycine max (L.) Merrill 1. *Crop Sci*](http://paperpile.com/b/QdsGZD/Ozm7). [1971; **11**: 929–931.](http://paperpile.com/b/QdsGZD/Ozm7)

5. [Gardes M, Bruns TD. ITS primers with enhanced specificity for basidiomycetes--application to the identification of mycorrhizae and rusts. *Mol Ecol*](http://paperpile.com/b/QdsGZD/48PI). [1993; **2**: 113–118.](http://paperpile.com/b/QdsGZD/48PI)

6. [Caporaso JG, Lauber CL, Walters WA, Berg-Lyons D, Lozupone CA, Turnbaugh PJ, et al. Global patterns of 16S rRNA diversity at a depth of millions of sequences per sample. *Proceedings of the National Academy of Sciences* . 2011; **108**: 4516–4522](http://paperpile.com/b/QdsGZD/CNhY).

7. [Caporaso JG, Kuczynski J, Stombaugh J, Bittinger K, Bushman FD, Costello EK, et al. QIIME allows analysis of high-throughput community sequencing data. *Nat Methods*](http://paperpile.com/b/QdsGZD/ZviC). [2010; **7**: 335–336.](http://paperpile.com/b/QdsGZD/ZviC)

8. [Martin M. Cutadapt removes adapter sequences from high-throughput sequencing reads. *EMBnet.journal* . 2011; **17**: 10](http://paperpile.com/b/QdsGZD/4GSD).

9. [Edgar RC. Search and clustering orders of magnitude faster than BLAST. *Bioinformatics*](http://paperpile.com/b/QdsGZD/x7HG). [2010; **26**: 2460–2461.](http://paperpile.com/b/QdsGZD/x7HG)

10. [Edgar RC. UPARSE: highly accurate OTU sequences from microbial amplicon reads. *Nat Methods*](http://paperpile.com/b/QdsGZD/E01p). [2013; **10**: 996–998.](http://paperpile.com/b/QdsGZD/E01p)

11. [Liber J, Bonito G, Benucci GMN. CONSTAX2: Improved taxonomic classification of environmental DNA markers.](http://paperpile.com/b/QdsGZD/6D96) *Bioinformatics*. 2021; btab347.

12. [Gdanetz K, Benucci GMN, Pol NV, Bonito G. CONSTAX: a tool for improved taxonomic resolution of environmental fungal ITS sequences. *BMC Bioinformatics* . 2017; **18**](http://paperpile.com/b/QdsGZD/ufZK).

13. [Website.](http://paperpile.com/b/QdsGZD/0nFE) [Abarenkov K, Zirk A, Piirmann T, Pöhönen R, Ivanov F, Nilsson RH, et al. UNITE general FASTA release for eukaryotes. Version 8.2 04.02.2020. UNITE Community. https://doi.org/10.15156/BIO/786370.](about:blank)

14. [Quast C, Pruesse E, Yilmaz P, Gerken J, Schweer T, Yarza P, et al. The SILVA ribosomal RNA gene database project: improved data processing and web-based tools. *Nucleic Acids Research*. 2012; **41**: D590–D596](http://paperpile.com/b/QdsGZD/Bhhe).

15. [Bowsher AW, Benucci GMN, Bonito G, Shade A. Seasonal Dynamics of Core Fungi in the Switchgrass Phyllosphere, and Co-Occurrence with Leaf Bacteria. *Phytobiomes Journal*. 2021; **5**: 60–68](http://paperpile.com/b/QdsGZD/gKuO).

16. [Zhang J, Liu Y-X, Zhang N, Hu B, Jin T, Xu H, et al. NRT1.1B is associated with root microbiota composition and nitrogen use in field-grown rice. *Nat Biotechnol*](http://paperpile.com/b/QdsGZD/xDpe). [2019; **37**: 676–684.](http://paperpile.com/b/QdsGZD/xDpe)

17. [Team RC, Others. R: A language and environment for statistical computing. 2013.](http://paperpile.com/b/QdsGZD/LUeU)

18. [McMurdie PJ, Holmes S. phyloseq: an R package for reproducible interactive analysis and graphics of microbiome census data. *PLoS One*](http://paperpile.com/b/QdsGZD/WA6X). [2013; **8**: e61217.](http://paperpile.com/b/QdsGZD/WA6X)

19. [Jari Oksanen FGB, Friendly M, Kindt R, Legendre P, McGlinn D, Minchin PR, et al. Vegan: community ecology package. *R package version*](http://paperpile.com/b/QdsGZD/kSOr). [2018; **2**.](http://paperpile.com/b/QdsGZD/kSOr)

20. [Davis NM, Proctor DM, Holmes SP, Relman DA, Callahan BJ. Simple statistical identification and removal of contaminant sequences in marker-gene and metagenomics data. *Microbiome*](http://paperpile.com/b/QdsGZD/udS7). [2018; **6**: 226.](http://paperpile.com/b/QdsGZD/udS7)

21. [Wickham H. ggplot2: Elegant Graphics for Data Analysis. (Springer Science & Business Media, 2009).](http://paperpile.com/b/QdsGZD/04mL)

22. [Paulson JN, Stine OC, Bravo HC, Pop M. Differential abundance analysis for microbial marker-gene surveys. *Nat Methods*](http://paperpile.com/b/QdsGZD/xmNQ). [2013; **10**: 1200–1202.](http://paperpile.com/b/QdsGZD/xmNQ)

**Supplemental Figures**


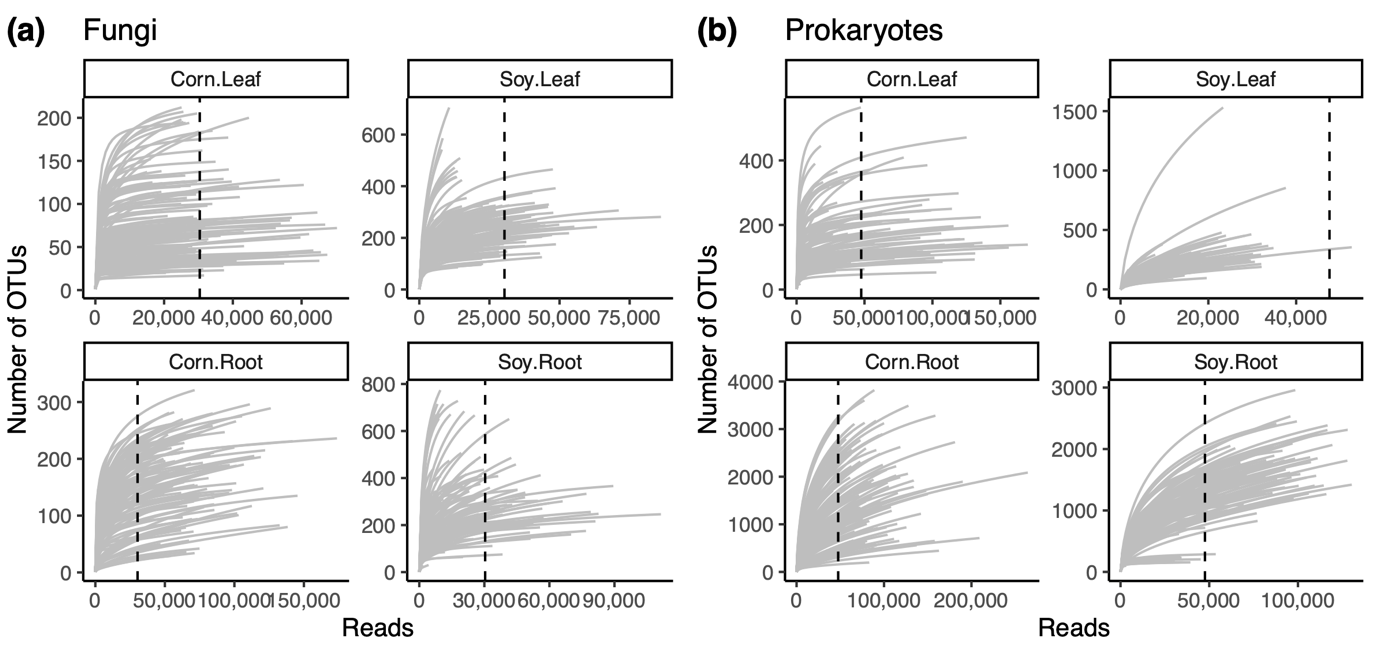


**Fig. S1.** Rarefaction curves for each sample sequenced in this study for (a) fungi and (b) prokaryotes in soybean or maize leaves and roots. The dashed line represents the median sequence depth.


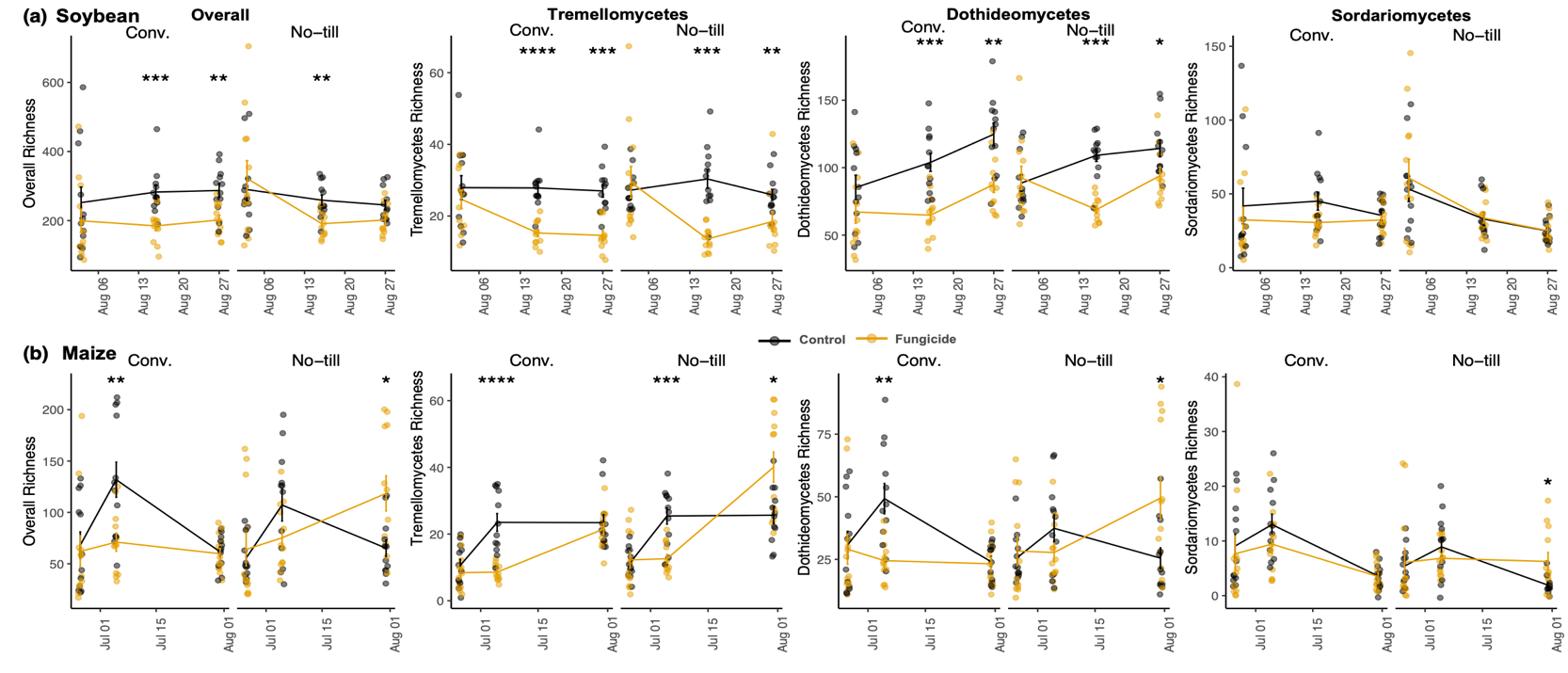


**Fig. S2.** Fungicidal effects on the richness of different fungal classes in soybean and maize phyllosphere. Black dots are control yellow dots are fungicide samples. Asterisks indicate the level of significance; * = p ≤ 0.05, ** = p ≤ 0.01, *** = p ≤ 0.001


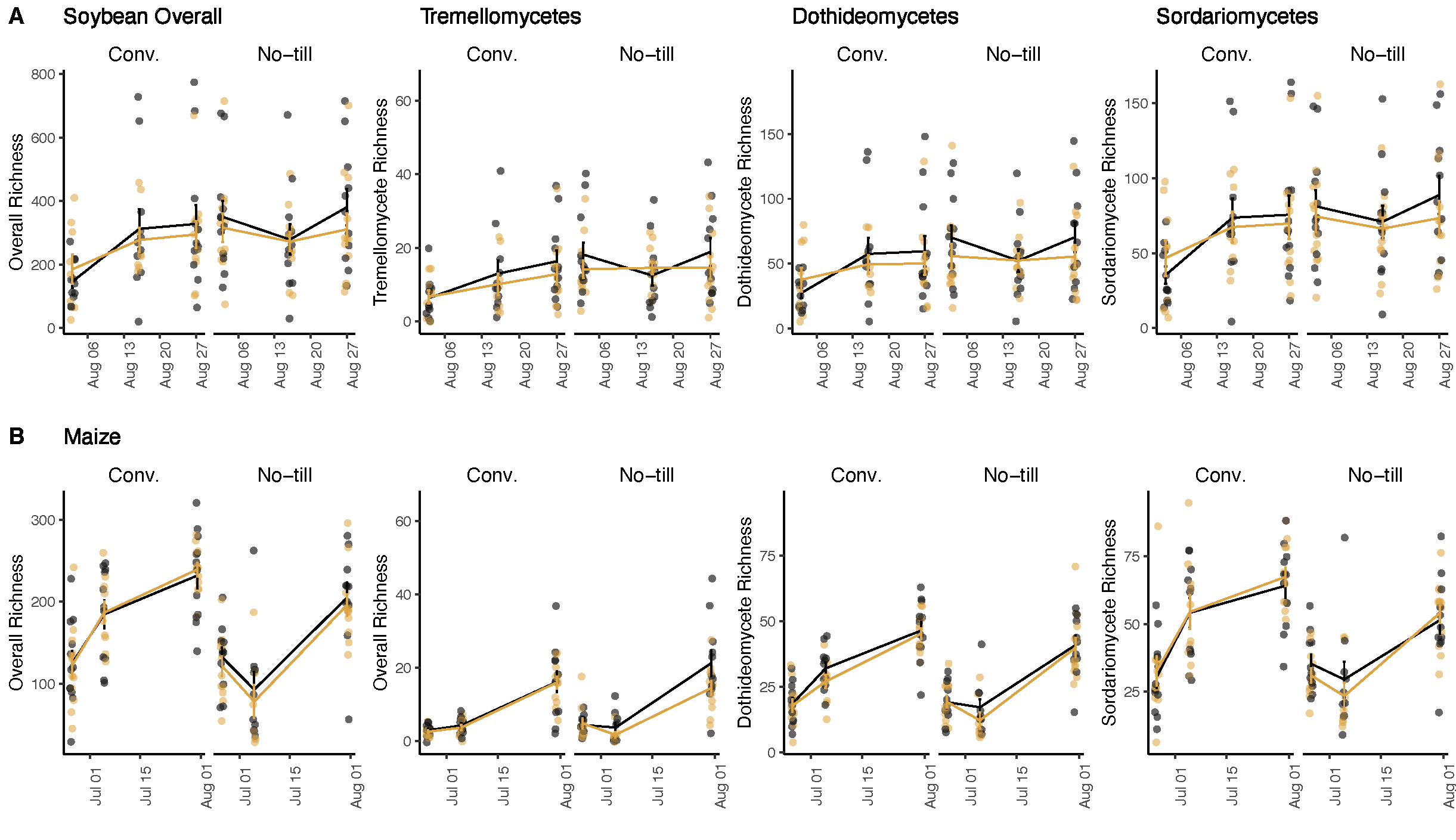


**Fig. S3.** Fungicidal effects on the richness of different fungal classes in soybean and maize roots. Black dots are control yellow dots are fungicide.


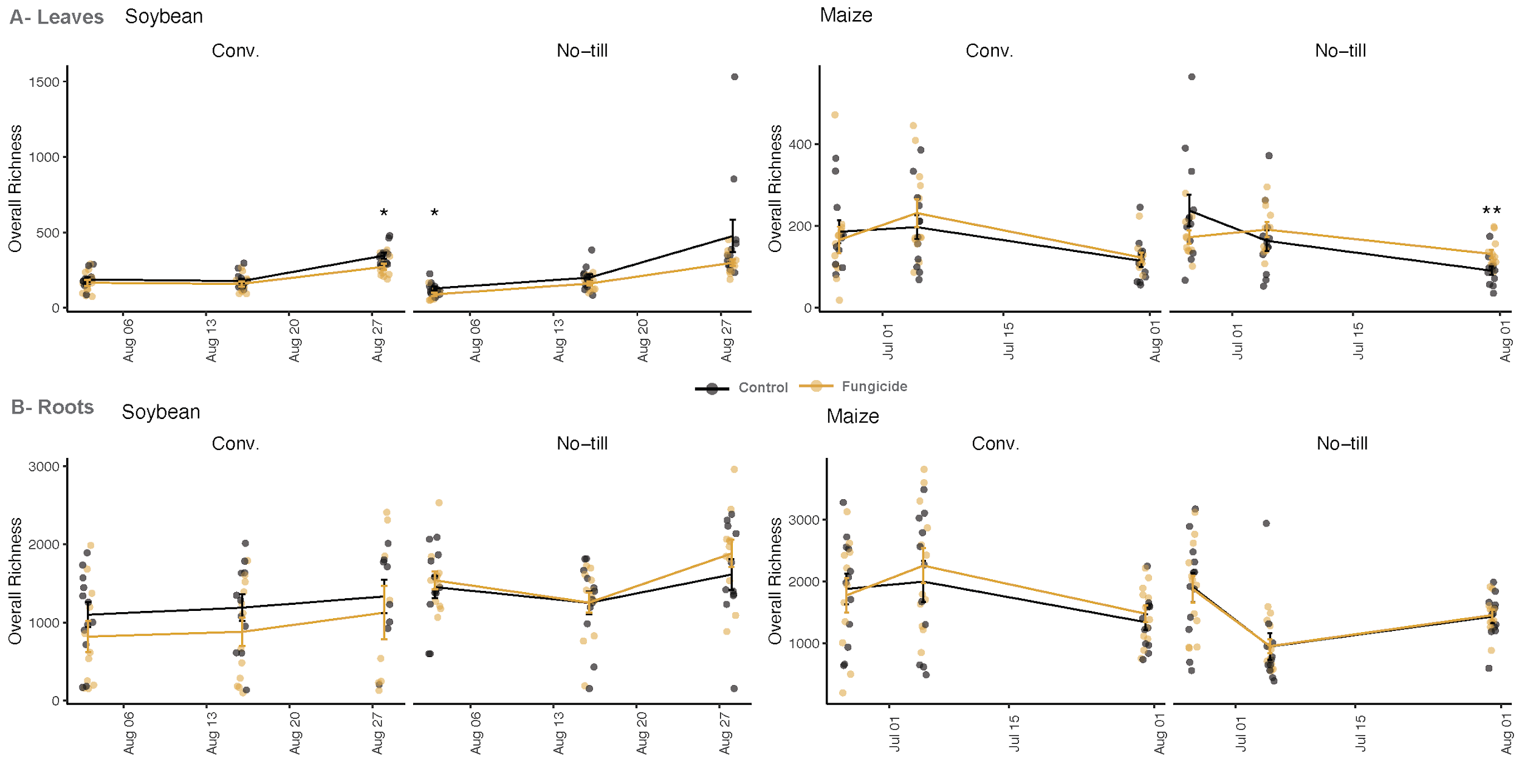


**Fig. S4.** Fungicidal effects on the richness of prokaryotes in soybean and maize roots and phyllospheres. Black dots are control yellow dots are fungicide. Asterisks indicate the level of significance; * = p ≤ 0.05, ** = p ≤ 0.01, *** = p ≤ 0.001.


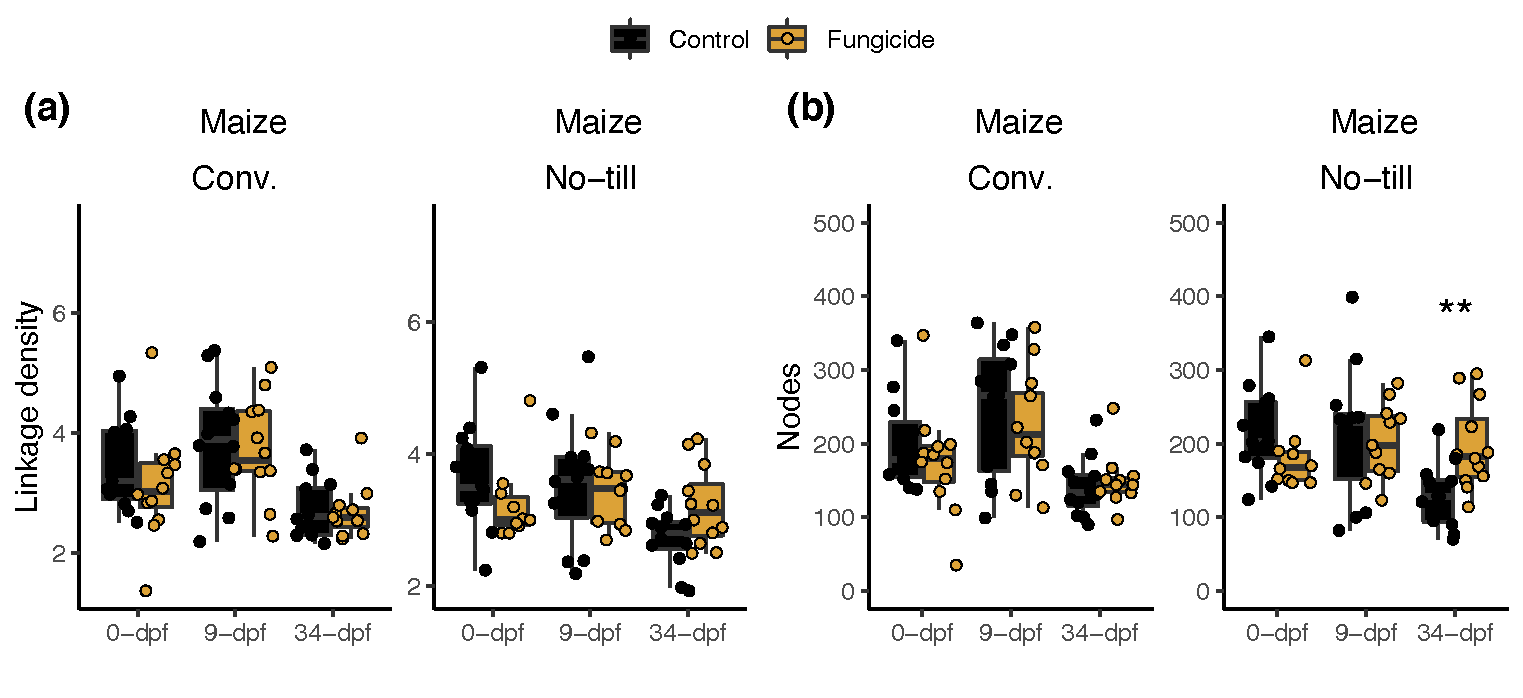


**Fig. S5.** A microbial co-occurrence network was constructed using taxa with a mean relative abundance greater than 10^-5^ and present in greater than 5 % of samples. Subnetworks were generated for each sample based on the OTUs present within those samples, and each point represents a subnetwork. (a) Network complexity (i.e., linkage density) and (b) number of edges were then calculated for each subnetwork. Comparisons are based on Wilcox ranked sign tests for maize conventional management and no-till. Asterisks indicate the level of significance; * = p ≤ 0.05, ** = p ≤ 0.01, *** = p ≤ 0.001.


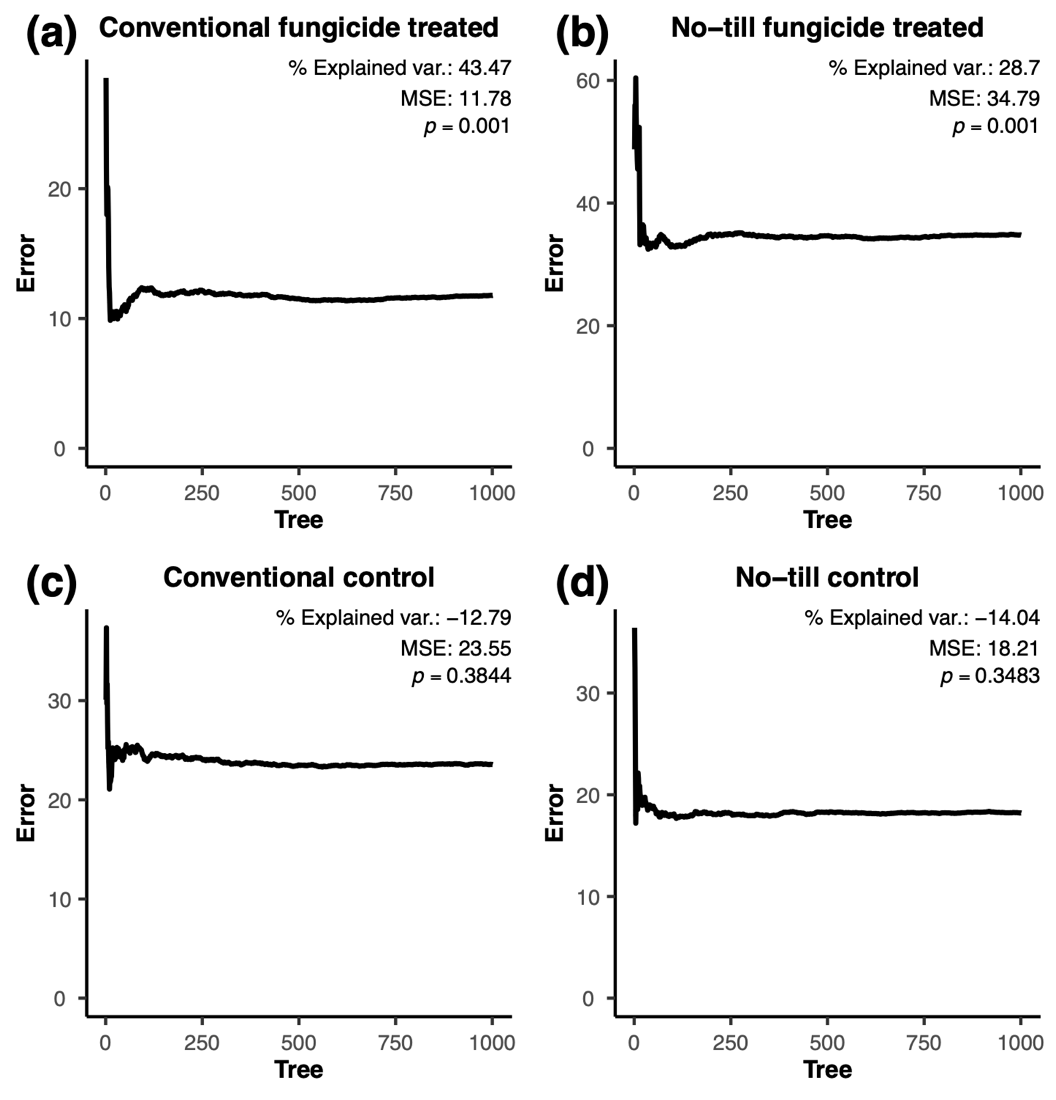


**Fig. S6.** Random forest models percent explained variance, error, and overall model significance (permutations = 999) for (a) conventional management treated with fungicides, (b) no-till treated with fungicides, (c) conventional management control, and (d) no-till control.

**Supplementary Tables**

**Table S1.** Three-step amplicon library preparation reagents and PCR master mixes

**Table S2.** Primers used for amplicon library preparation

**Table S3.** Cycling conditions for PCR in library preparation for fungi and prokaryotes

**Table S4.** Permutational multivariate analysis of variance for fungi in maize or soybean in roots or leaves before and after fungicide application

**Table S5.** Permutational multivariate analysis of variance for prokaryotes in maize or soybean in roots or leaves before and after fungicide application

**Table S6.** Effects of fungicide on maize and soybean leaf fungal composition

**Table S7.** Differentially abundant phyllosphere fungal OTUs by fungicide treatment – provided as separate file

**Table S8.** Core members of the soybean or maize phyllosphere in no-till and conventional management – provided separate file

**Table S9.** Recovery status of fungicide-impacted soybean phyllosphere fungal OTUs – provided as separate file
